# Scalar and vector self-energies of heavy baryons in nuclear medium

**DOI:** 10.1016/j.nuclphysa.2017.02.007

**Published:** 2017-04

**Authors:** K. Azizi, N. Er, H. Sundu

**Affiliations:** a Department of Physics, Doǧuş University, Acıbadem-Kadıköy, 34722 Istanbul, Turkey; b Department of Physics, Abant İzzet Baysal University, Gölköy Kampüsü, 14980 Bolu, Turkey; c Department of Physics, Kocaeli University, 41380 İzmit, Turkey

**Keywords:** Nuclear matter, Heavy hadrons, Mass, Residue, Scalar and vector self-energies

## Abstract

The in-medium sum rules are employed to calculate the shifts in the mass and residue as well as the scalar and vector self-energies of the heavy $\Lambda_{Q},\Sigma_{Q}$ and $\Xi_{Q}$ baryons, with *Q* being *b* or *c* quark. The maximum shift in mass due to nuclear matter belongs to the $\Sigma_{c}$ baryon and it is found to be $\Delta m_{\Sigma_{c}}=-936\;\text{MeV}$. In the case of residue, it is obtained that the residue of $\Sigma_{b}$ baryon is maximally affected by the nuclear medium with the shift $\Delta \lambda_{\Sigma_{b}}=-0.014\;{\text{GeV}}^{3}$. The scalar and vector self-energies are found to be $\Sigma_{\Lambda_{b}}^{S}=653\;\text{MeV}$, $\Sigma_{\Sigma_{b}}^{S}=-614\;\text{MeV}$, $\Sigma_{\Xi_{b}}^{S}=-17\;\text{MeV}$, $\Sigma_{\Lambda_{c}}^{S}=272\;\text{MeV}$, $\Sigma_{\Sigma_{c}}^{S}=-936\;\text{MeV}$, $\Sigma_{\Xi_{c}}^{S}=-5\;\text{MeV}$ and $\Sigma_{\Lambda_{b}}^{\nu }=436\pm 148\;\text{MeV}$, $\Sigma_{\Sigma_{b}}^{\nu }=382\pm 129\;\text{MeV}$, $\Sigma_{\Xi_{b}}^{\nu }=15\pm 5\;\text{MeV}$, $\Sigma_{\Lambda_{c}}^{\nu }=151\pm 45\;\text{MeV}$, $\Sigma_{\Sigma_{c}}^{\nu }=486\pm 144\;\text{MeV}$ and $\Sigma_{\Xi_{c}}^{\nu }=1.391\pm 0.529\;\text{MeV}$.

## 1 Introduction

The investigation of the in-medium properties of hadrons constituents one of the main directions of the research in high energy and particle physics both theoretically and experimentally. Such studies can help us not only better understand the structure of the hot and dense astrophysical objects like the neutron stars and analyze the results of the heavy ion collision experiments, but also get valuable knowledge on the perturbative and nonperturbative aspects of QCD and the nature of the quark–gluon plasma as the new phase of matter. From the experimental side, the PANDA and CBM Collaborations at FAIR aim to study the in-medium properties of not only the standard but also the non-conventional exotic states newly discovered by different collaborations [1], [2], [3], [4], [5], [6], [7].

From the theoretical side, it can be found many studies devoted to the in-medium properties of the light and heavy mesons as well as the light baryons (see for instance [8], [9], [10], [11], [12], [13], [14], [15], [16], [17], [18], [19], [20], [21]). In the heavy baryon sector, however, there are few works dedicated to the investigation of the spectroscopic parameters of the heavy baryons in nuclear medium [22], [23], [24]. In these studies, the authors use the Ioffe current to study some properties of heavy and doubly heavy baryons.

In the present study, we use the interpolating currents with an arbitrary mixing parameter to investigate the shifts in the mass and pole residue of the heavy $\Lambda_{Q},\Sigma_{Q}$ and $\Xi_{Q}$ baryons due to the nuclear medium. We employ the in-medium QCD sum rule approach to calculate also the scalar and vector self-energies of those baryons by fixing the mixing parameter entered the interpolating currents according to the standard prescriptions. We shall note that we do not analyze the heavy $\Omega_{Q}$ baryon here since the nuclear matter considered in the present work does not affect the parameters of the $\Omega_{Q}$ baryon considerably as it does not contain the *u* or *d* quark. One may investigate the shifts on the parameters of this baryon in hyperonic and strange matters or nuclear medium with strange component.

This work is organized as follows. In section 2, we obtain the in-medium QCD sum rules for mass and residue as well as the scalar and vector self-energies of the heavy $\Lambda_{Q},\Sigma_{Q}$ and $\Xi_{Q}$ baryons in nuclear matter. In section 3, the numerical analyses of sum rules are performed and the results are compared with those of the vacuum and other predictions obtained via Ioffe currents in the literature. The last section contains our concluding remarks. Some lengthy expressions obtained during calculations are moved to the Appendix A.

## 2 Mass, residue and self-energies of heavy baryons in nuclear matter

In order to calculate the mass, residue as well as the scalar and vector self-energies of the heavy baryons using the QCD sum rule method in nuclear medium, the first step is to construct an in-medium two-point correlation function:

(1) $$\Pi =i\int d^{4}xe^{ip\cdot x}\langle \psi_{0}|T[J_{B_{Q}}(x){\overset{¯}{J}}_{B_{Q}}(0)]|\psi_{0}\rangle$$

where *p* is the four momentum of the heavy baryon, $|\psi_{0}\rangle$ is the nuclear matter ground state and $J_{B_{Q}}$ is the interpolating current of the heavy $B_{Q}=\Lambda_{Q},\Sigma_{Q},\Xi_{Q}$ baryons. These interpolating currents are given as

(2) $$\begin{array}{ccc} J_{\Sigma_{Q}} & = & -\frac{1}{\sqrt{2}}\epsilon^{abc}\{(q_{1}^{aT}CQ^{b})\gamma_{5}q_{2}^{c}+\beta (q_{1}^{aT}C\gamma_{5}Q^{b})q_{2}^{c}\\ & & -[(Q^{aT}Cq_{2}^{b})\gamma_{5}q_{1}^{c}+\beta (Q^{aT}C\gamma_{5}q_{2}^{b})q_{1}^{c}]\},\\ J_{\Lambda_{Q},\Xi_{Q}} & = & \frac{1}{\sqrt{6}}\epsilon^{abc}\{2(q_{1}^{aT}Cq_{2}^{b})\gamma_{5}Q^{c}+2\beta (q_{1}^{aT}C\gamma_{5}q_{2}^{b})Q^{c}\\ & & +(q_{1}^{aT}CQ^{b})\gamma_{5}q_{2}^{c}+\beta (q_{1}^{aT}C\gamma_{5}Q^{b})q_{2}^{c}\\ & & +(Q^{aT}Cq_{2}^{b})\gamma_{5}q_{1}^{c}+\beta (Q^{aT}C\gamma_{5}q_{2}^{b})q_{1}^{c}\} \end{array}$$

where a,b,c are color indices and *C* is the charge conjugation operator. The parameter *β* is an arbitrary auxiliary parameter with β=−1 being corresponding to the Ioffe current, which is obtained after a Fierz transformation [25] (for more details on the interpolating fields see [25], [26], [27], [28]). The quark fields $q_{1}$ and $q_{2}$ for different members are given in Table 1.

In accordance with the standard philosophy of the method used, we shall calculate the aforesaid correlation function in hadronic and operator product expansion (OPE) sides. Matching the coefficients of different structures from these two sides through a dispersion relation leads to the sum rules for the different observables. In the hadronic side a complete set of heavy $B_{Q}$ baryon with the same quantum numbers as the interpolating currents is inserted into the correlation function. After performing integral over four-x we get

(3) $$\Pi^{Had}=-\frac{\langle \psi_{0}|J_{B_{Q}}(0)|B_{Q}(p^{⁎},s)\rangle \langle B_{Q}(p^{⁎},s)|{\overset{¯}{J}}_{B_{Q}}(0)|\psi_{0}\rangle }{p^{⁎2}-m_{B_{Q}}^{⁎2}}+...,$$

where $|B_{Q}(p^{⁎},s)\rangle$ is the heavy baryon state with spin *s* and in-medium momentum $p^{⁎}$ and ... represents the contributions of the higher states and the continuum. In the above equation, $m_{Q}^{⁎}$ is the modified mass of the heavy baryon in medium. The matrix elements seen in the above equation can be parametrized as

(4) $$\begin{array}{ccc} \langle \psi_{0}|J_{B_{Q}}(0)|B_{Q}(p^{⁎},s)\rangle & = & \lambda_{B_{Q}}^{⁎}u_{B_{Q}}(p^{⁎},s)\\ \langle B_{Q}(p^{⁎},s)|{\overset{¯}{J}}_{B_{Q}}(0)|\psi_{0}\rangle & = & {\overset{¯}{\lambda }}_{B_{Q}}^{⁎}{\overset{¯}{u}}_{B_{Q}}(p^{⁎},s) \end{array}$$

where $\lambda_{B_{Q}}^{⁎}$ is the modified residue or the coupling strengths of the heavy baryon to nuclear medium and $u_{B_{Q}}(p^{⁎},s)$ is the in-medium Dirac spinor. Using Eq. (4) in Eq. (3) and summing over the spins of the heavy baryon, the hadronic side of the in-medium correlation function can be written as

(5) $$\begin{array}{ccc} \Pi^{Had} & = & -\frac{\lambda_{B_{Q}}^{⁎2}({\mathrm{p̸}}^{⁎}+m_{B_{Q}}^{⁎})}{p^{⁎2}-m_{B_{Q}}^{⁎2}}+...=-\frac{\lambda_{B_{Q}}^{⁎2}}{{\mathrm{p̸}}^{⁎}-m_{B_{Q}}^{⁎}}+...\\ & = & -\frac{\lambda_{B_{Q}}^{⁎2}}{\left(p_{B_{Q}}^{\mu }-\Sigma_{\nu B_{Q}}^{\mu })\gamma_{\mu }-(m_{B_{Q}}+\Sigma_{B_{Q}}^{S}\right)}+..., \end{array}$$

where $\Sigma_{\nu B_{Q}}^{\mu }$ and $\Sigma_{B_{Q}}^{S}$ are the vector and scalar self-energies of the heavy baryon in nuclear matter, respectively [29]. The vector self-energy can be written as

(6) $$\Sigma_{\nu B_{Q}}^{\mu }=\Sigma_{B_{Q}}^{\nu }u^{\mu }+\Sigma_{\nu B_{Q}}^{\prime }p_{B_{Q}}^{\mu },$$

where $u^{\mu }$ is the four velocity of the nuclear medium and we ignore $\Sigma_{\nu B_{Q}}^{\prime }$ because of its small value [30]. The four-velocity of the nuclear matter creates extra structures to the correlation function compared to the vacuum QCD sum rules. The calculations are performed in the rest frame of the nuclear medium, i.e. $u^{\mu }=(1,0)$. After the substitution of Eq. (6) into Eq. (5), we get the hadronic side of the correlation function in terms of three different structures as

(7) $$\Pi^{Had}=\Pi_{p}^{Had}(p^{2},p_{0})\mathrm{p̸}+\Pi_{u}^{Had}(p^{2},p_{0})\mathrm{u̸}+\Pi_{S}^{Had}(p^{2},p_{0})I+...,$$

here $p_{0}$ is the energy of the quasi-particle, *I* is the unit matrix and

(8) $$\begin{array}{ccc} \Pi_{p}^{Had}(p^{2},p_{0}) & = & -\lambda_{B_{Q}}^{⁎2}\frac{1}{p^{2}-\mu_{B_{Q}}^{2}},\\ \Pi_{u}^{Had}(p^{2},p_{0}) & = & +\lambda_{B_{Q}}^{⁎2}\frac{\Sigma_{B_{Q}}^{\nu }}{p^{2}-\mu_{B_{Q}}^{2}},\\ \Pi_{S}^{Had}(p^{2},p_{0}) & = & -\lambda_{B_{Q}}^{⁎2}\frac{m_{B_{Q}}^{⁎}}{p^{2}-\mu_{B_{Q}}^{2}}, \end{array}$$

where $m_{B_{Q}}^{⁎}=m_{B_{Q}}+\Sigma_{B_{Q}}^{S}$ and $\mu_{B_{Q}}^{2}=m_{B_{Q}}^{⁎2}-\Sigma_{B_{Q}}^{\nu 2}+2p_{0}\Sigma_{B_{Q}}^{\nu }$. After applying the Borel transformation with respect to $p^{2}$, we obtain

(9) $$\begin{array}{ccc} \overset{ˆ}{B}\Pi_{p}^{Had}(p^{2},p_{0}) & = & \lambda_{B_{Q}}^{⁎2}e^{-\mu_{B_{Q}}^{2}/M^{2}},\\ \overset{ˆ}{B}\Pi_{u}^{Had}(p^{2},p_{0}) & = & -\lambda_{B_{Q}}^{⁎2}\Sigma_{\nu B_{Q}}e^{-\mu_{B_{Q}}^{2}/M^{2}},\\ \overset{ˆ}{B}\Pi_{S}^{Had}(p^{2},p_{0}) & = & \lambda_{B_{Q}}^{⁎2}m_{B_{Q}}^{⁎}e^{-\mu_{B_{Q}}^{2}/M^{2}}, \end{array}$$

where $M^{2}$ is the Borel mass parameter to be fixed in the next section.

The QCD representation of the correlation function is derived in the deep Euclidean region. The correlator in this side is also decomposed in terms of the selected structures as

(10) $$\Pi^{QCD}=\Pi_{p}^{QCD}(p^{2},p_{0})\mathrm{p̸}+\Pi_{u}^{QCD}(p^{2},p_{0})\mathrm{u̸}+\Pi_{S}^{QCD}(p^{2},p_{0})I.$$

To calculate the correlation function in QCD side, we insert the interpolating currents (2) into the correlation function (1) and perform the contractions of the quark pair using the Wick's theorem. As a result, we get expressions presented in the Appendix A for different particles in terms of the heavy and light quark propagators.

To proceed, we need to know the expressions for the quark propagators in the coordinate space. In the fixed point gauge, the light and heavy quark propagators are chosen as

(11) $$\begin{array}{ccc} S_{q}^{ij}(x) & = & \frac{i}{2\pi^{2}}\delta^{ij}\frac{1}{{\left(x^{2}\right)}^{2}}\mathrm{x̸}-\frac{m_{q}}{4\pi^{2}}\delta^{ij}\frac{1}{x^{2}}+\chi_{q}^{i}(x){\overset{¯}{\chi }}_{q}^{j}(0)\\ & & -\frac{ig_{s}}{32\pi^{2}}F_{\mu \nu }^{A}(0)t^{ij,A}\frac{1}{x^{2}}[\mathrm{x̸}\sigma^{\mu \nu }+\sigma^{\mu \nu }\mathrm{x̸}]+\cdots \;,\\ S_{Q}^{ij}(x) & = & \frac{i}{{\left(2\pi \right)}^{4}}\int d^{4}ke^{-ik\cdot x}\{\frac{\delta_{ij}}{\mathrm{k̸}-m_{Q}}-\frac{g_{s}F_{\mu \nu }^{A}(0)t^{ij,A}}{4}\frac{\sigma_{\mu \nu }(\mathrm{k̸}+m_{Q})+(\mathrm{k̸}+m_{Q})\sigma_{\mu \nu }}{{\left(k^{2}-m_{Q}^{2}\right)}^{2}}\\ & & +\frac{\pi^{2}}{3}\langle \frac{\alpha_{s}GG}{\pi }\rangle \delta_{ij}m_{Q}\frac{k^{2}+m_{Q}\mathrm{k̸}}{{\left(k^{2}-m_{Q}^{2}\right)}^{4}}+\cdots \}\;, \end{array}$$

where $\chi_{q}^{i}$ and ${\overset{¯}{\chi }}_{q}^{j}$ are the Grassmann background quark fields, $F_{\mu \nu }^{A}$ are classical background gluon fields, and $t^{ij,A}=\frac{\lambda^{ij,A}}{2}$ with $\lambda^{ij,A}$ being the standard Gell-Mann matrices. The quark, gluon and mixed condensates are defined in terms of different operators in nuclear matter. To avoid from redundancy, we do not present their explicit expressions here, but refer the readers to Refs. [16], [30] for more details.

The QCD side of the correlation function in the Borel scheme can be written in terms of the perturbative and nonperturbative parts as

(12) $$\begin{array}{ccc} \overset{ˆ}{B}\Pi_{p,u,S}^{QCD}(p^{2},p_{0}) & = & \overset{ˆ}{B}\Pi_{p,u,S}^{Pert}(p^{2},p_{0})+\overset{ˆ}{B}\Pi_{p,u,S}^{qq}(p^{2},p_{0})+\overset{ˆ}{B}\Pi_{p,u,S}^{GG}(p^{2},p_{0})\\ & & +\overset{ˆ}{B}\Pi_{p,u,S}^{qGq}(p^{2},p_{0})+\overset{ˆ}{B}\Pi_{p,u,S}^{qqqq}(p^{2},p_{0}), \end{array}$$

where Pert represents the perturbative part and the upper indices *qq*, *GG*, *qGq* and qqqq denote the contributions of two-quark, two-gluon, mixed and four-quark condensates, respectively. Writing each function in terms of the even and odd parts as $\overset{ˆ}{B}\Pi_{j}^{i}(p^{2},p_{0})=\overset{ˆ}{B}\Pi_{j,E}^{i}(p^{2},p_{0})+p_{0}\overset{ˆ}{B}\Pi_{j,O}^{i}(p^{2},p_{0})$, for the structure *p̸* and $\Lambda_{Q}$ baryon we obtain

(13) $$\begin{array}{ccc} \overset{ˆ}{B}\Pi_{p,E}^{Pert}(p^{2},p_{0}) & = & \frac{1}{256\pi^{4}}(1+\beta )(5+\beta )\\ & & \times \int_{m_{Q}^{2}}^{s_{0}}\{m_{Q}^{8}-8m_{Q}^{6}s+8m_{Q}^{2}s^{3}-s^{4}+12m_{Q}^{4}s^{2}Log[m_{Q}^{2}/s]\}\frac{e^{-\frac{s}{M^{2}}}}{s^{2}}ds,\\ \overset{ˆ}{B}\Pi_{p,O}^{Pert}(p^{2},p_{0}) & = & 0,\\ \overset{ˆ}{B}\Pi_{p,E}^{qq}(p^{2},p_{0}) & = & \frac{1}{216\pi^{2}}\int_{m_{Q}^{2}}^{s_{0}}\{-{\left(m_{Q}^{2}-s\right)}^{2}[(9(1+(2-3\beta )\beta )m_{Q}s\\ & & +2(1+\beta )(38+7\beta )m_{q}(2m_{Q}^{2}+s)){\langle \overset{¯}{u}u\rangle }_{\rho_{N}}\\ & & +(2i(1+\beta )(17+\beta ){\left(m_{Q}^{2}-s\right)}^{2}(2m_{Q}^{2}-s)){\langle u^{†}iD_{0}u\rangle }_{\rho_{N}}\\ & & -{\left(m_{Q}^{2}-s\right)}^{2}(9(1+(2-3\beta )\beta )m_{Q}s\\ & & +2(1+\beta )(38+7\beta )m_{q}(2m_{Q}^{2}+s)){\langle \overset{¯}{d}d\rangle }_{\rho_{N}}\\ & & +(2i(1+\beta )(17+\beta ){\left(m_{Q}^{2}-s\right)}^{2}(2m_{Q}^{2}-s)){\langle d^{†}iD_{0}d\rangle }_{\rho_{N}}]\}\frac{e^{-\frac{s}{M^{2}}}}{s^{3}}ds,\\ \overset{ˆ}{B}\Pi_{p,O}^{qq}(p^{2},p_{0}) & = & \frac{1}{216\pi^{2}}\int_{m_{Q}^{2}}^{s_{0}}\{(6(1+\beta )(7+2\beta ){\left(m_{Q}^{2}-s\right)}^{2}(2m_{Q}^{2}-s)){\langle u^{†}u\rangle }_{\rho_{N}}\\ & & +(6(1+\beta )(7+2\beta ){\left(m_{Q}^{2}-s\right)}^{2}(2m_{Q}^{2}-s)){\langle d^{†}d\rangle }_{\rho_{N}}\}\frac{e^{-\frac{s}{M^{2}}}}{s^{3}}ds,\\ \overset{ˆ}{B}\Pi_{p,E}^{GG}(p^{2},p_{0}) & = & \frac{1}{36864\pi^{4}}{\left(1+\beta \right)}^{2}\\ & & \times \int_{m_{Q}^{2}}^{s_{0}}\{(m_{Q}^{2}-s)[125m_{Q}^{4}+152m_{Q}^{2}s+107s^{2}]{\langle \frac{\alpha_{s}}{\pi }G^{2}\rangle }_{\rho_{N}}\}\frac{e^{-\frac{s}{M^{2}}}}{s^{3}}ds,\\ \overset{ˆ}{B}\Pi_{p,O}^{GG}(p^{2},p_{0}) & = & 0,\\ \overset{ˆ}{B}\Pi_{p,E(O)}^{qGq}(p^{2},p_{0}) & = & 0, \end{array}$$

where $s_{0}$ is the continuum threshold and we do not show the even and odd parts of $\overset{ˆ}{B}\Pi_{p,u,S}^{qqqq}(p^{2},p_{0})$ here because of their very lengthy expressions. The four-quark condensate is poorly known in the nuclear medium. Hence, by the help of a density dependent factor, it is factorized into multiplication of two two-quark condensates. The value of this factor is determined by examination of its effect on predictions from QCD sum rules (for details see [25]). As the impact of this factor is very weak in the case of heavy baryons we use the naive factorizations [30]

(14) $${\langle q_{a\alpha }{\overset{¯}{q}}_{b\beta }q_{c\gamma }{\overset{¯}{q}}_{d\delta }\rangle }_{\rho_{N}}≃{\langle q_{a\alpha }{\overset{¯}{q}}_{b\beta }\rangle }_{\rho_{N}}{\langle q_{c\gamma }{\overset{¯}{q}}_{d\delta }\rangle }_{\rho_{N}}-{\langle q_{a\alpha }{\overset{¯}{q}}_{d\delta }\rangle }_{\rho_{N}}{\langle q_{c\gamma }{\overset{¯}{q}}_{b\beta }\rangle }_{\rho_{N}},$$

in the case of same quarks and

(15) $${\langle q_{a\alpha }{\overset{¯}{q}}_{b\beta }q_{c\gamma }^{\prime }{\overset{¯}{q}}_{d\delta }^{\prime }\rangle }_{\rho_{N}}≃{\langle q_{a\alpha }{\overset{¯}{q}}_{b\beta }\rangle }_{\rho_{N}}{\langle q_{c\gamma }^{\prime }{\overset{¯}{q}}_{d\delta }^{\prime }\rangle }_{\rho_{N}}$$

for different quarks as well as the expansion [30],

(16) 〈qaα(x)q¯bβ(0)〉ρN=−δab12[(〈q¯q〉ρN+xμ〈q¯Dμq〉ρN+12xμxν〈q¯DμDνq〉ρN+...)δαβ〈qaα(x)q¯bβ(0)〉ρN=+(〈q¯γλq〉ρN+xμ〈q¯γλDμq〉ρN+12xμxν〈q¯γλDμDνq〉ρN+...)γαβλ],

in terms of the operators having 3,4 and 5 mass dimensions in nuclear matter. We ignore from the contributions of the more than four-quark and two-gluon operators as their values are unknown at finite density (for more details see for instance [30]).

As a final goal, the coefficients of three structures from both hadronic and QCD sides are matched. This leads to the following sum rules:

(17) $$\begin{array}{ccc} \lambda_{B_{Q}}^{⁎2}e^{-\mu_{B_{Q}}^{2}/M^{2}} & = & \overset{ˆ}{B}\Pi_{p}^{QCD},\\ -\lambda_{B_{Q}}^{⁎2}\Sigma_{B_{Q}}^{\nu }e^{-\mu_{B_{Q}}^{2}/M^{2}} & = & \overset{ˆ}{B}\Pi_{u}^{QCD},\\ \lambda_{B_{Q}}^{⁎2}m_{B_{Q}}^{⁎}e^{-\mu_{B_{Q}}^{2}/M^{2}} & = & \overset{ˆ}{B}\Pi_{S}^{QCD}. \end{array}$$

By solving these equations and an extra equation, that is obtained by applying a derivative to both sides of the first equation with respect to $-1/M^{2}$, simultaneously we find the parameters $\lambda_{B_{Q}}^{⁎},m_{B_{Q}}^{⁎},\Sigma_{B_{Q}}^{\nu }$ and $\mu_{B_{Q}}$ as

(18) $$\begin{array}{ccc} \mu_{B_{Q}}^{2}(s_{0},M^{2},\beta ) & = & \frac{d\overset{ˆ}{B}\Pi_{p}^{QCD}/d(-1/M^{2})}{\overset{ˆ}{B}\Pi_{p}^{QCD}},\\ \lambda_{B_{Q}}^{⁎2}(s_{0},M^{2},\beta ) & = & e^{\mu_{B_{Q}}^{2}/M^{2}}\overset{ˆ}{B}\Pi_{p}^{QCD},\\ \Sigma_{B_{Q}}^{\nu }(s_{0},M^{2},\beta ) & = & -\frac{\overset{ˆ}{B}\Pi_{u}^{QCD}}{\overset{ˆ}{B}\Pi_{p}^{QCD}},\\ m_{B_{q}}^{⁎}(s_{0},M^{2},\beta ) & = & \frac{\overset{ˆ}{B}\Pi_{S}^{QCD}}{\overset{ˆ}{B}\Pi_{p}^{QCD}}. \end{array}$$

Alternatively, we can use the previously introduced relation,

(19) $$m_{B_{q}}^{⁎2}(s_{0},M^{2},\beta )=\mu_{B_{Q}}^{2}+\Sigma_{B_{Q}}^{\nu 2}-2p_{0}\Sigma_{B_{Q}}^{\nu },$$

to find the mass of the baryons under consideration at finite density.

Here we should remark that by the method used for the in-medium heavy baryons in the present study including the Borel transformation with respect to $p^{2}$ and the chosen continuum threshold we automatically extracted the contributions of the particles with positive energy excitations. This can be considered as an alternative to the canonical approach to in-medium QCD sum rules for nucleons [30], [31], which uses Cauchy's theorem in the $p_{0}$ plane with a cut along the full $Im[p_{0}]=0$ axis thus including necessarily positive and negative frequencies at fixed $\overset{\to }{p}$. For the light baryons especially the nucleons which contain exactly the same quark ingredients with the medium one shall consider both the particle and antiparticle contributions at fixed three-momentum of the particles and separate the contributions of the positive frequency particles by the help of an appropriate weight function (for details see also [25]).

## 3 Numerical results and discussion

The numerical analyses of the QCD sum rules for the mass, pole residue, scalar and vector self-energies of the heavy $\Lambda_{Q},\Sigma_{Q}$ and $\Xi_{Q}$ in nuclear medium, require the values of quark and baryon masses, nuclear matter density, different in-medium quark, gluon and mixed condensates, etc. Their numerical values are collected in Table 2.

Note that we use the average value y=0.05 to perform the numerical analyses.

Besides the above input parameters, the sum rules obtained for the mass, residue and self-energies of heavy baryons contain three more auxiliary parameters that should be fixed: the Borel parameter $M^{2}$, the continuum threshold $s_{0}$ and the mixing parameter *β*. The standard way in determining the working regions of these parameters is that the physical observables demonstrate weak dependence on them. The continuum threshold is not totally arbitrary but it depends on the energy of the first excited state with the same quantum numbers. This parameter is related to the beginning of the continuum in the channels under consideration. If the ground state mass is given by $m_{B_{Q}}$, $\sqrt{s_{0}}-m_{B_{Q}}$ is the energy needed to excite the particle to its first excited state. According to the standard prescriptions and considering the masses of the first excited states in the channels under study, we choose this energy in the interval 0.1 GeV–0.4 GeV, which leads to the following intervals for the *b*-baryons under consideration:

(20) $$\begin{array}{c} 32.7\;{\text{GeV}}^{2}⩽s_{0}^{\Lambda_{b}}⩽36.2\;{\text{GeV}}^{2},\\ 34.9\;{\text{GeV}}^{2}⩽s_{0}^{\Sigma_{b}}⩽38.6\;{\text{GeV}}^{2},\\ 34.7\;{\text{GeV}}^{2}⩽s_{0}^{\Xi_{b}}⩽38.3\;{\text{GeV}}^{2}; \end{array}$$

for the *c*-baryons we get

(21) $$\begin{array}{c} 5.7\;{\text{GeV}}^{2}⩽s_{0}^{\Lambda_{c}}⩽7.2\;{\text{GeV}}^{2},\\ 6.5\;{\text{GeV}}^{2}⩽s_{0}^{\Sigma_{c}}⩽8.1\;{\text{GeV}}^{2},\\ 6.6\;{\text{GeV}}^{2}⩽s_{0}^{\Xi_{c}}⩽8.2\;{\text{GeV}}^{2}. \end{array}$$

Our analyses show that in these intervals, the dependence of the physical parameters on the continuum threshold is weak. The standard criteria in calculating the Borel window are that not only the pole contribution exceeds the contributions of the higher states and continuum but also the series of OPE converge, i.e. the higher operators contribute less to the total integral compared to the lower operators and the perturbative contribution overcomes to the nonperturbative ones. These criteria lead to the following intervals:

(22) $$5\;{\text{GeV}}^{2}⩽M^{2}⩽8\;{\text{GeV}}^{2}\;\;\;\;\;\text{for}\;\;\Lambda_{b},\Sigma_{b},\Xi_{b},$$

and

(23) $$3\;{\text{GeV}}^{2}⩽M^{2}⩽6\;{\text{GeV}}^{2}\;\;\;\;\;\text{for}\;\;\Lambda_{c},\Sigma_{c},\Xi_{c}.$$

The pole dominance together with the aforesaid intervals for the Borel and threshold parameters leads to the following windows to the parameter *β*

(24) −0.6⩽x⩽−0.4   and   0.4⩽x⩽0.6,

where we used x=cos⁡θ with $\theta =\tan^{-1}\beta$ notation to explore the whole region from −∞ to +∞ by varying *x* in the interval [−1,1]. Note that the Ioffe current with x=−0.71 remains out of the reliable regions in our calculations. Our numerical calculations show that in the above regions the results relatively show weak dependence on the *x* or *β*.

**Fig. 1:**
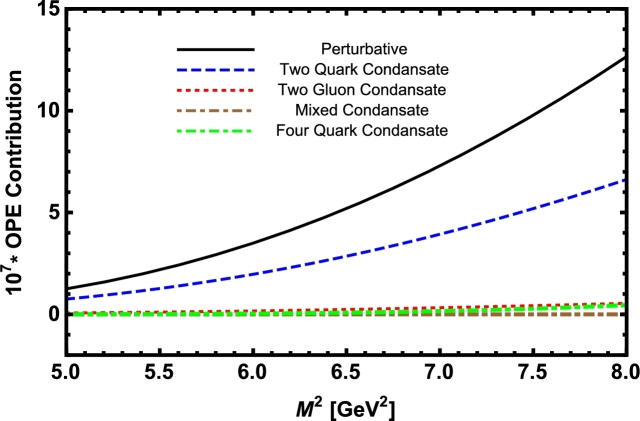
Variations of perturbative part, two-quark condensate, two-gluon condensate, mixed condensate and four-quark condensate in Λ_*b*_ channel and *p̸* structure with respect to *M*^2^ at average values of *s*_0_ and *x*.

**Fig. 2:**
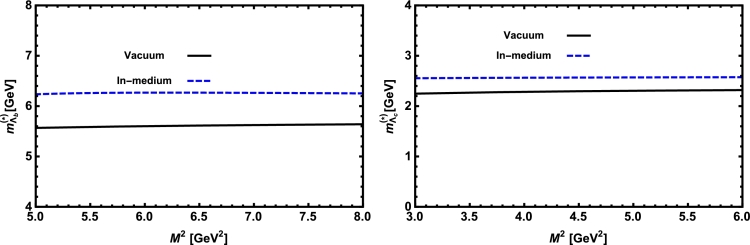
The mass of the Λ_*b*_ baryon (left panel) and the Λ_*c*_ baryon (right panel) versus *M*^2^ in vacuum and nuclear medium at average values of *s*_0_ and *x*.

**Fig. 3:**
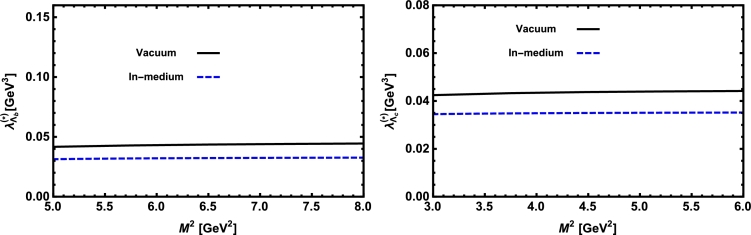
The residue of the Λ_*b*_ baryon (left panel) and the Λ_*c*_ baryon (right panel) versus *M*^2^ in vacuum and nuclear medium at average values of *s*_0_ and *x*.

**Fig. 4:**
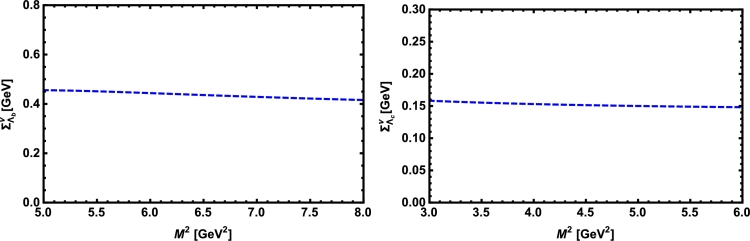
The vector self-energy of the Λ_*b*_ baryon (left panel) and the Λ_*c*_ baryon (right panel) versus *M*^2^ in nuclear medium at average values of *s*_0_ and *x*.

**Fig. 5:**
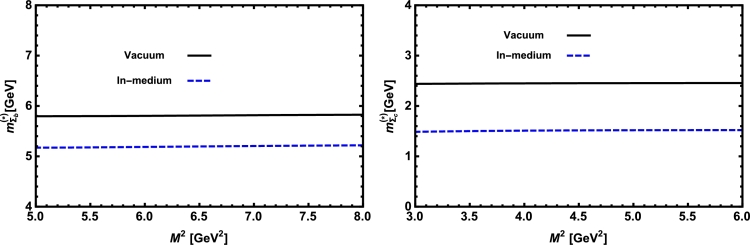
The mass of the Σ_*b*_ baryon (left panel) and the Σ_*c*_ baryon (right panel) versus *M*^2^ in vacuum and nuclear medium at average values of *s*_0_ and *x*.

**Fig. 6:**
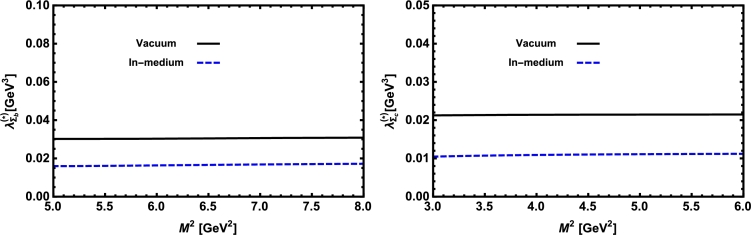
The residue of the Σ_*b*_ baryon (left panel) and the Σ_*c*_ baryon (right panel) versus *M*^2^ in vacuum and nuclear medium at average values of *s*_0_ and *x*.

**Fig. 7:**
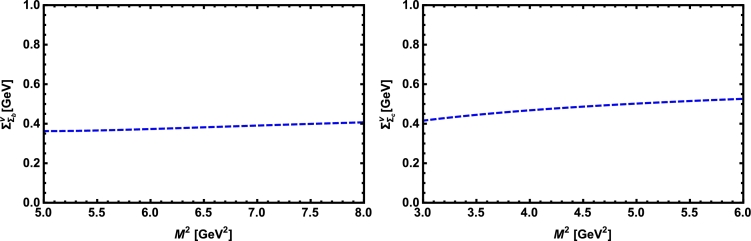
The vector self-energy of the Σ_*b*_ baryon (left panel) and the Σ_*c*_ baryon (right panel) versus *M*^2^ in nuclear medium at average values of *s*_0_ and *x*.

**Fig. 8:**
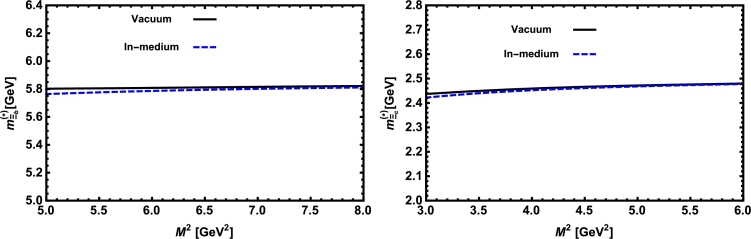
The mass of the Ξ_*b*_ baryon (left panel) and the Ξ_*c*_ baryon (right panel) versus *M*^2^ in vacuum and nuclear medium at average values of *s*_0_ and *x*.

**Fig. 9:**
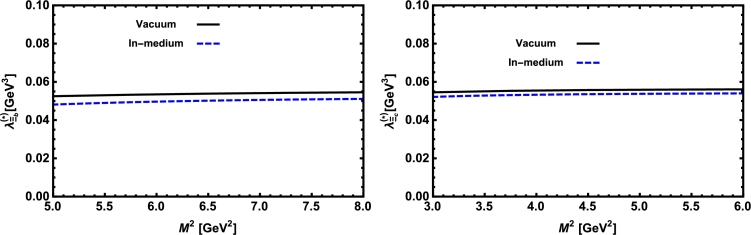
The residue of the Ξ_*b*_ baryon (left panel) and the Ξ_*c*_ baryon (right panel) versus *M*^2^ in vacuum and nuclear medium at average values of *s*_0_ and *x*.

**Fig. 10:**
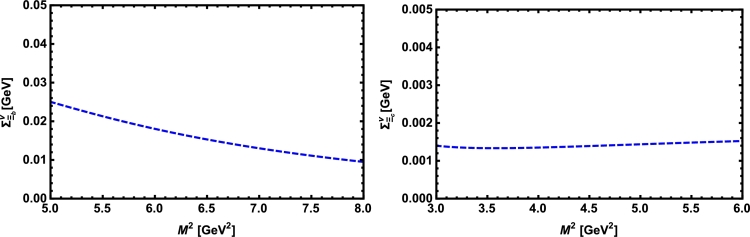
The vector self-energy of the Ξ_*b*_ baryon (left panel) and the Ξ_*c*_ baryon (right panel) versus *M*^2^ in nuclear medium at average values of *s*_0_ and *x*.

Now, we proceed to numerically analyze the sum rules obtained for the physical observables using these working windows and the values of other input parameters. To this end, first of all, in order to show how the OPE converges in our calculations we compare the variations of perturbative part, two-quark condensate, two-gluon condensate, mixed condensate and four-quark condensate with approximations in Eqs. (14) and (15) for instance in $\Lambda_{b}$ channel and *p̸* structure with respect to $M^{2}$ at average values of other auxiliary parameters in Fig. 1. From this figure, we see that the OPE nicely converges, i.e., the perturbative part exceeds the nonperturbative contributions and the contributions reduce with increasing the dimension. Note that as is also clear from Eq. (13) the contribution of mixed condensate is exactly zero since the terms containing the mixed condensate have no imaginary parts and do not contribute to the spectral density. We also see that the four-quark condensate has least contribution to the sum rules and the approximations (14) and (15) seem reasonable. Similar results are obtained for other channels and structures. We plot the quantities under consideration, i.e. masses, residues, the vector self-energies with respect to $M^{2}$ at average values of the threshold and mixing parameters in Fig. 2, Fig. 3, Fig. 4, Fig. 5, Fig. 6, Fig. 7, Fig. 8, Fig. 9, Fig. 10 for both the nuclear medium and vacuum. Only in the case of vector self-energies we depict their variations with respect to the Borel parameter for different particles in medium since they do not exist in the vacuum. We shall also remark that we calculate the scalar self-energies via the shifts in masses compared to their vacuum values, hence we do not plot their variations with respect to $M^{2}$, separately. First of all, we see that these figures depict considerable shifts on the values of observables due to nuclear matter when we compare them with their vacuum values. The next issue that should be emphasized is: the physical observables under consideration overall show weak dependence on the Borel parameter both in vacuum and nuclear medium in the selected windows. Extracted from the numerical calculations, we present the average numerical results for different quantities for both the nuclear matter and vacuum and also both the *b* and *c*-baryons in Table 3, Table 4, Table 5. The quoted errors in the values are due to the uncertainties coming from the calculations of the working regions of auxiliary parameters, errors of different input parameters as well as those related to different approximations used in the analyses. We present the existing predictions for parameters in $\Lambda_{Q}$ and $\Sigma_{Q}$ channels obtained via Ioffe current [22], [23] in Table 3, Table 4 as well.

A quick glance at these tables leads to the following results:

• the values of masses obtained in vacuum ($m_{B_{Q}}$) are in nice consistencies with the experimental data presented in Table 2 and borrowed from PDG [32]. The results of vacuum residues $\lambda_{B_{Q}}$ are also consistent with the predictions of, for instance, Ref. [38] obtained by employing the vacuum sum rules.
• The values for the masses and residues obtained in nuclear medium ($m_{B_{Q}}^{⁎}$ and $\lambda_{B_{Q}}^{⁎}$) show considerably large shifts from their vacuum values. These shifts are large in $\Lambda_{Q}$ and $\Sigma_{Q}$ channels compared to the $\Xi_{Q}$ baryon. This is an expected situation since $\Lambda_{Q}$ and $\Sigma_{Q}$ baryons contain both the up and down quarks interacting with nuclear matter of the same quark content, while the $\Xi_{Q}$ baryon includes only one of them and the strange quark.
• The maximum and minimum shifts in the masses due to nuclear medium belong to $\Sigma_{c}$ and $\Xi_{c}$ baryons, respectively. The maximum and minimum deviations of the in-medium residues from their vacuum values belong to $\Sigma_{b}$ and $\Xi_{c}$ baryons, respectively.
• The maximum vector self-energy belongs to the $\Sigma_{c}$ baryon, while its minimum value corresponds to $\Xi_{c}$ channel.
• The mass shifts in $\Lambda_{b,c}$ channels are positive against the negative mass shifts in $\Sigma_{b,c}$ and $\Xi_{b,c}$ channels. The sign of shifts in the residues for all channels are negative.
• Although our results for the vacuum masses in $\Lambda_{Q}$ channel are consistent with those of [22], our predictions for the vacuum masses $m_{\Sigma_{b}}$ and $m_{\Sigma_{c}}$ differ considerably with those of [23]. The $\Sigma_{Q}$ baryon masses obtained in [23] in the $\rho_{N}\to 0$ limit are much smaller than the experimental data due to the unequal pole residues from different structures considered in this work, therefore the authors normalize the masses to the experimental data to obtain the scalar self-energies presented in Table 4.
• The in-medium results for masses, residues and self-energies as well as the vacuum residues overall show large discrepancies between our predictions and those of [22], [23]. This can be attributed to the different working regions of auxiliary parameters used in [22], [23]. We remind that the Ioffe current used in these works remains outside of the reliable region for the mixing parameter entered to the currents in our case. When comparing the sign of the self-energies in our work and the existing predictions from [22], [23] we see that the vector and scalar self-energies have the same signs in these studies.
• The amount of shifts due to nuclear medium in our case is overall large compared to the results of [22], [23] in $\Lambda_{Q}$ and $\Sigma_{Q}$ channels. In [22] and $\Lambda_{Q}$ channel some parameters like residues have not even been affected by the medium noticeably.
• Comparison of the results with those of Q=s (hyperons) analyzed in Ref. [17] reveals that the shifts in the scalar and vector self-energies in $\Sigma_{Q}$ and $\Lambda_{Q}$ channels are grater than those of the hyperons. In Ξ channel, however, we see an inverse situation.

**Fig. 11:**
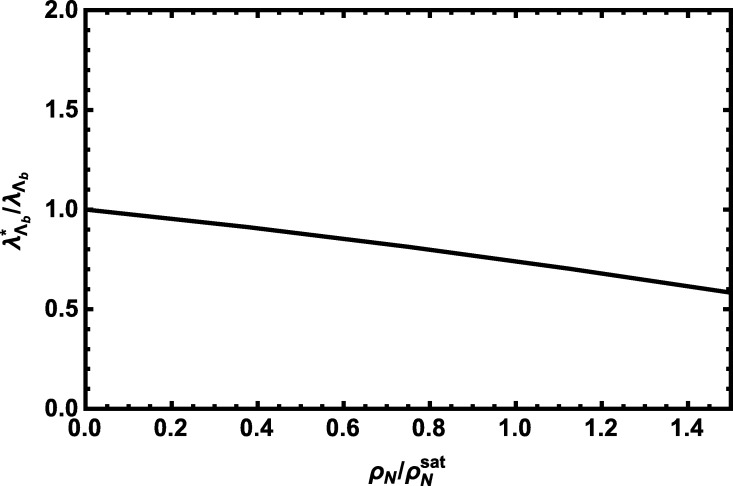
$\lambda_{\Lambda_{b}}^{⁎}/\lambda_{\Lambda_{b}}$ versus $\rho_{N}/\rho_{N}^{sat}$ at the average values of auxiliary parameters.

At the end of this section, we would like to discuss the density dependence of the results. Note that in above analyses we have used the linear density approximation in operators listed in Table 2 and the saturation density $\rho_{N}^{sat}={\left(0.11\right)}^{3}\;{\text{GeV}}^{3}$ to obtain the numerical results. As an example, we plot $\lambda_{\Lambda_{b}}^{⁎}/\lambda_{\Lambda_{b}}$ versus $\rho_{N}/\rho_{N}^{sat}$ at the average values of auxiliary parameters in Fig. 11. $\lambda_{\Lambda_{b}}^{⁎}/\lambda_{\Lambda_{b}}$ better represents the density-dependence of the OPE for the structure *p̸* (see Eq. (18)) normalized by a constant, i.e. $\lambda_{\Lambda_{b}}$ (residue in vacuum) as it is proportional to the function $\overset{ˆ}{B}\Pi_{p}^{QCD}$ not any ratio of two OPE expressions. From this figure we see that the dependence of the quantity $\lambda_{\Lambda_{b}}^{⁎}/\lambda_{\Lambda_{b}}$ on density is roughly linear and it decreases with the increasing density, considerably.

## 4 Conclusion

In the present study, using the interpolating currents with an arbitrary mixing parameter, the in-medium sum rules are utilized to calculate the shifts in the mass and residue as well as the scalar and vector self-energies of the heavy $\Lambda_{Q},\Sigma_{Q}$ and $\Xi_{Q}$ baryons. Imposing the conditions of OPE convergence and pole dominance, we found working regions of the auxiliary parameters entered the calculations. The obtained results reveal that the shifts in the masses of $\Lambda_{b,c}$ are found to be positive, while the shits in the residues of these baryons as well as the shifts in the masses and residues of the $\Sigma_{b,c}$ and $\Xi_{b,c}$ baryons are obtained to be negative. Both the *b* and *c* baryons are considerably affected by the medium. The maximum shift in mass due to nuclear medium corresponds to the $\Sigma_{c}$ baryon with the value $\Delta m_{\Sigma_{c}}=-936\;\text{MeV}$. In the case of residue, it is obtained that the residue of $\Sigma_{b}$ baryon is maximally affected by the nuclear medium with the shift $\Delta \lambda_{\Sigma_{b}}=-0.014\;{\text{GeV}}^{3}$. The scalar and vector self-energies are obtained to be $\Sigma_{\Lambda_{b}}^{S}=653\;\text{MeV}$, $\Sigma_{\Sigma_{b}}^{S}=-614\;\text{MeV}$, $\Sigma_{\Xi_{b}}^{S}=-17\;\text{MeV}$, $\Sigma_{\Lambda_{c}}^{S}=272\;\text{MeV}$, $\Sigma_{\Sigma_{c}}^{S}=-936\;\text{MeV}$, $\Sigma_{\Xi_{c}}^{S}=-5\;\text{MeV}$ and $\Sigma_{\Lambda_{b}}^{\nu }=436\pm 148\;\text{MeV}$, $\Sigma_{\Sigma_{b}}^{\nu }=382\pm 129\;\text{MeV}$, $\Sigma_{\Xi_{b}}^{\nu }=15\pm 5\;\text{MeV}$, $\Sigma_{\Lambda_{c}}^{\nu }=151\pm 45\;\text{MeV}$, $\Sigma_{\Sigma_{c}}^{\nu }=486\pm 144\;\text{MeV}$ and $\Sigma_{\Xi_{c}}^{\nu }=1.391\pm 0.529\;\text{MeV}$. Our results in the limit of $\rho_{N}\to 0$ are in a good consistency with the experimental data and existing theoretical predictions. We observed that, because of their quark contents, the $\Xi_{Q}$ baryons are minimally affected by the nuclear matter of up and down quarks in comparison with the $\Lambda_{Q}$ and $\Sigma_{Q}$ baryons. Comparison of the results with those of hyperons analyzed in Ref. [17] reveals that the shifts in the scalar and vector self-energies of $\Sigma_{Q}$ and $\Lambda_{Q}$ baryons are grater than those of the hyperons against the Ξ channel, at which the situation is inverse.

We performed our numerical analyses and obtained the results in Table 3, Table 4, Table 5 using the linear density approximation for different operators entered to the numerical calculations (see Table 2) and the saturation density $\rho_{N}^{sat}={\left(0.11\right)}^{3}\;{\text{GeV}}^{3}$. We discussed the dependence of the results, as an example $\lambda_{\Lambda_{b}}^{⁎}/\lambda_{\Lambda_{b}}$ for the structure *p̸*, on the nuclear density and found the dependence is roughly linear and the quantity $\lambda_{\Lambda_{b}}^{⁎}/\lambda_{\Lambda_{b}}$ decreases by increasing the density, considerably.

Within the working regions of the auxiliary parameters the OPE nicely converges, the perturbative part exceeds the nonperturbative one and the main nonperturbative contribution comes from the two-quark condensate. The two-gluon, mixed and poorly known four-quark condensates have very small impacts on the numerical results in the case of heavy baryons.

Looking at the obtained numerical results in the present study at zero temperature (cold nuclear matter) we conclude that the shifts in the parameters of the $\Sigma_{Q}$ and $\Lambda_{Q}$ for both heavy *b* and *c* quarks due to nuclear matter are considerably large and deserve experimental investigations by different collaborations. The shift in the central value of the mass of the $\Sigma_{c}$ with 38% is maximal. The shift in the residue of $\Sigma_{b}$ channel with amount of 45% is the highest among the shifts in the central values of residues. The vector self-energies are also obtained to be considerably large and experimentally measurable in these channels. The parameters of $\Xi_{Q}$ with both heavy quarks, however, do not show considerably large shifts due to nuclear matter. If we combine these results with the results of Ref. [17] on the parameters of hyperons, which also show considerably large shifts due to nuclear medium especially in the case of negative parity hyperons, we can say that the shifts in the parameters of both heavy and light spin-1/2 baryons are overall remarkably large and experimentally measurable. These results may shed light to the PANDA and CBM experiments, which envisage to study the hyperons and charmed baryons in T=0 nuclear matter. The results of the present study can be checked via different phenomenological approaches as well. To compare the results with those of the heavy ion collision experiments, one needs to add the effects of finite temperature to the results obtained in the present study.

## Figures and Tables

**Table 1:** The quark flavors *q*_1_ and *q*_2_ for the baryons under consideration.

	$\Lambda_{b(c)}^{0(+)}$	$\Sigma_{b(c)}^{0(+)}$	$\Xi_{b(c)}^{0(+)}$
*q*_1_	*u*	*u*	*u*
*q*_2_	*d*	*d*	*s*

**Table 2:** Numerical values for input parameters. Note that we use the average value *y* = 0.05 to perform the numerical analyses.

Input parameters	Values
*p*_0_	$m_{B_{Q}}$
*m*_*u*_; *m*_*d*_; *m*_*s*_	$2.3_{-0.5}^{0.7}\;\text{MeV}$; $4.83_{-0.3}^{0.5}\;\text{MeV}$; 95 ± 5 MeV [32]
*m*_*b*_; *m*_*c*_	4.78 ± 0.06 GeV; 1.275 ± 0.025 GeV [32]
$m_{\Lambda_{b}}$; $m_{\Lambda_{c}}$	5619.51 ± 0.23 MeV; 2286.46 ± 0.14 MeV [32]
$m_{\Sigma_{b}}$; $m_{\Sigma_{c}}$	5811.3 ± 1.9 MeV; 2452.9 ± 0.4 MeV [32]
$m_{\Xi_{b}}$; $m_{\Xi_{c}}$	5791.8 ± 0.5 MeV; $2467.93_{-0.40}^{+0.28}\;\text{MeV}$[32]
*ρ*_*N*_	(0.11)^3^ GeV^3^[33]
${\langle q^{†}q\rangle }_{\rho_{N}}$; ${\langle s^{†}s\rangle }_{\rho_{N}}$	$\frac{3}{2}\rho_{N}$; 0 [33]
${\langle \overset{¯}{q}q\rangle }_{0}$; ${\langle \overset{¯}{s}s\rangle }_{0}$	(−0.241)^3^ GeV^3^; $0.8\;{\langle \overset{¯}{q}q\rangle }_{0}$[34], [35]
*m*_*q*_	0.5(*m*_*u*_ + *m*_*d*_) [33]
*σ*_*N*_; $\sigma_{N_{0}}$	0.045 GeV; 0.035 GeV [33]
*y*	0.04 ± 0.02 [36]; 0.066 ± 0.011 ± 0.002 [37]
${\langle \overset{¯}{q}q\rangle }_{\rho_{N}}$; ${\langle \overset{¯}{s}s\rangle }_{\rho_{N}}$	${\langle \overset{¯}{q}q\rangle }_{0}+\frac{\sigma_{N}}{2m_{q}}\rho_{N}$; ${\langle \overset{¯}{s}s\rangle }_{0}+y\frac{\sigma_{N}}{2m_{q}}\rho_{N}$[33]
${\langle q^{†}g_{s}\sigma Gq\rangle }_{\rho_{N}}$; ${\langle s^{†}g_{s}\sigma Gs\rangle }_{\rho_{N}}$	−0.33 GeV^2^*ρ*_*N*_; −*y*0.33 GeV^2^*ρ*_*N*_[33]
${\langle q^{†}iD_{0}q\rangle }_{\rho_{N}}$; ${\langle s^{†}iD_{0}s\rangle }_{\rho_{N}}$	0.18 GeV*ρ*_*N*_; $\frac{m_{s}{\langle \overset{¯}{s}s\rangle }_{\rho_{N}}}{4}+0.02\;\text{GeV}\rho_{N}$[33]
${\langle \overset{¯}{q}iD_{0}q\rangle }_{\rho_{N}}$; ${\langle \overset{¯}{s}iD_{0}s\rangle }_{\rho_{N}}$	$\frac{3}{2}m_{q}\rho_{N}≃0$; 0 [33]
$m_{0}^{2}$	0.8 GeV^2^[34], [35]
${\langle \overset{¯}{q}g_{s}\sigma Gq\rangle }_{0}$; ${\langle \overset{¯}{s}g_{s}\sigma Gs\rangle }_{0}$	$m_{0}^{2}{\langle \overset{¯}{q}q\rangle }_{0}$; $m_{0}^{2}{\langle \overset{¯}{s}s\rangle }_{0}$
${\langle \overset{¯}{q}g_{s}\sigma Gq\rangle }_{\rho_{N}}$; ${\langle \overset{¯}{s}g_{s}\sigma Gs\rangle }_{\rho_{N}}$	${\langle \overset{¯}{q}g_{s}\sigma Gq\rangle }_{0}+3\;{\text{GeV}}^{2}\rho_{N}$; ${\langle \overset{¯}{s}g_{s}\sigma Gs\rangle }_{0}+3y\;{\text{GeV}}^{2}\rho_{N}$[33]
${\langle \overset{¯}{q}iD_{0}iD_{0}q\rangle }_{\rho_{N}}$; ${\langle \overset{¯}{s}iD_{0}iD_{0}s\rangle }_{\rho_{N}}$	$0.3\;{\text{GeV}}^{2}\rho_{N}-\frac{1}{8}{\langle \overset{¯}{q}g_{s}\sigma Gq\rangle }_{\rho_{N}}$; $0.3y\;{\text{GeV}}^{2}\rho_{N}-\frac{1}{8}{\langle \overset{¯}{s}g_{s}\sigma Gs\rangle }_{\rho_{N}}$[33]
${\langle q^{†}iD_{0}iD_{0}q\rangle }_{\rho_{N}}$; ${\langle s^{†}iD_{0}iD_{0}s\rangle }_{\rho_{N}}$	$0.031\;{\text{GeV}}^{2}\rho_{N}-\frac{1}{12}{\langle q^{†}g_{s}\sigma Gq\rangle }_{\rho_{N}}$; $0.031y\;{\text{GeV}}^{2}\rho_{N}-\frac{1}{12}{\langle s^{†}g_{s}\sigma Gs\rangle }_{\rho_{N}}$[33]
${\langle \frac{\alpha_{s}}{\pi }G^{2}\rangle }_{0}$	(0.33 ± 0.04)^4^ GeV^4^[34], [35]
${\langle \frac{\alpha_{s}}{\pi }G^{2}\rangle }_{\rho_{N}}$	${\langle \frac{\alpha_{s}}{\pi }G^{2}\rangle }_{0}-0.65\;\text{GeV}\rho_{N}$[33]

**Table 3:** The numerical values of residues, masses in vacuum and nuclear matter, vector self-energies in nuclear matter and scalar self-energies of Λ_*b*_ and Λ_*c*_ baryons.

	$\lambda_{\Lambda_{b}}$ [GeV^3^]	$\lambda_{\Lambda_{b}}^{⁎}$ [GeV^3^]	$m_{\Lambda_{b}}$ [GeV]	$m_{\Lambda_{b}}^{⁎}$ [GeV]	$\Sigma_{\Lambda_{b}}^{\nu }$ [MeV]	$\Sigma_{\Lambda_{b}}^{S}$ [MeV]
Present study	0.044 ± 0.008	0.032 ± 0.006	5.614 ± 0.345	6.267 ± 0.175	436 ± 148	653
Ioffe current [22]	0.027 ± 0.003	0.026 ± 0.003	$5.618_{-0.104}^{+0.078}$	$5.678_{-0.103}^{+0.077}$	32 ± 1	60


	$\lambda_{\Lambda_{c}}$ [GeV^3^]	$\lambda_{\Lambda_{c}}^{⁎}$ [GeV^3^]	$m_{\Lambda_{c}}$ [GeV]	$m_{\Lambda_{c}}^{⁎}$ [GeV]	$\Sigma_{\Lambda_{c}}^{\nu }$ [MeV]	$\Sigma_{\Lambda_{c}}^{S}$ [MeV]
Present study	0.044 ± 0.012	0.035 ± 0.010	2.295 ± 0.251	2.567 ± 0.362	151 ± 45	272
Ioffe current [22]	0.022 ± 0.002	0.021 ± 0.001	$2.284_{-0.078}^{+0.049}$	$2.335_{-0.072}^{+0.045}$	34 ± 1	51

**Table 4:** The numerical values of residues, masses in vacuum and nuclear matter, vector self-energies in nuclear matter and scalar self-energies of Σ_*b*_ and Σ_*c*_ baryons.

	$\lambda_{\Sigma_{b}}$ [GeV^3^]	$\lambda_{\Sigma_{b}}^{⁎}$ [GeV^3^]	$m_{\Sigma_{b}}$ [GeV]	$m_{\Sigma_{b}}^{⁎}$ [GeV]	$\Sigma_{\Sigma_{b}}^{\nu }$ [MeV]	$\Sigma_{\Sigma_{b}}^{S}$ [MeV]
Present study	0.031 ± 0.010	0.017 ± 0.005	5.810 ± 0.241	5.196 ± 0.667	382 ± 129	−614
Ioffe current [23]	$8.73_{-0.65}^{+0.90}\times 10^{-3}$	$1.25_{-0.04}^{+0.08}\times 10^{-2}$	$3.56_{-0.10}^{+0.14}$	$3.33_{-0.07}^{+0.09}$	$776_{-35}^{+42}$	−375


	$\lambda_{\Sigma_{c}}$ [GeV^3^]	$\lambda_{\Sigma_{c}}^{⁎}$ [GeV^3^]	$m_{\Sigma_{c}}$ [GeV]	$m_{\Sigma_{c}}^{⁎}$ [GeV]	$\Sigma_{\Sigma_{c}}^{\nu }$ [MeV]	$\Sigma_{\Sigma_{c}}^{S}$ [MeV]
Present study	0.021 ± 0.007	0.011 ± 0.004	2.451 ± 0.208	1.515 ± 0.321	486 ± 144	−936
Ioffe current [23]	$1.99_{-0.26}^{+0.29}\times 10^{-2}$	$2.46_{-0.16}^{+0.22}\times 10^{-2}$	$1.40_{-0.05}^{+0.08}$	$1.33_{-0.03}^{+0.06}$	$446_{-27}^{+35}$	−123

**Table 5:** The numerical values of residues, masses in vacuum and nuclear matter, vector self-energies in nuclear matter and scalar self-energies of Ξ_*b*_ and Ξ_*c*_ baryons.

	$\lambda_{\Xi_{b}}$ [GeV^3^]	$\lambda_{\Xi_{b}}^{⁎}$ [GeV^3^]	$m_{\Xi_{b}}$ [GeV]	$m_{\Xi_{b}}^{⁎}$ [GeV]	$\Sigma_{\Xi_{b}}^{\nu }$ [MeV]	$\Sigma_{\Xi_{b}}^{S}$ [MeV]
Present study	0.054 ± 0.012	0.050 ± 0.018	5.812 ± 0.179	5.795 ± 0.127	15 ± 5	−17


	$\lambda_{\Xi_{c}}$ [GeV^3^]	$\lambda_{\Xi_{c}}^{⁎}$ [GeV^3^]	$m_{\Xi_{c}}$ [GeV]	$m_{\Xi_{c}}^{⁎}$ [GeV]	$\Sigma_{\Xi_{c}}^{\nu }$ [MeV]	$\Sigma_{\Xi_{c}}^{S}$ [MeV]
Present study	0.056 ± 0.021	0.054 ± 0.020	2.466 ± 0.118	2.461 ± 0.130	1.391 ± 0.529	−5

## Appendix A Correlation functions in terms of quark propagators for different heavy baryons

In this appendix, we collect the results for correlation functions in QCD side obtained after insertion of the interpolating currents and contracting out the quark fields in OPE side. For the function $\Pi_{\Lambda_{b}}^{OPE}$ we get

(A.1) $$\begin{array}{ccc} \Pi_{\Lambda_{b}}^{OPE} & = & -\frac{i}{6}\epsilon_{abc}\epsilon_{a^{\prime }b^{\prime }c^{\prime }}\int d^{4}xe^{ipx}\langle \psi_{0}|\{(2\gamma_{5}S_{b}^{ca^{\prime }}(x)S_{u}^{\prime \;ab^{\prime }}(x)S_{d}^{bc^{\prime }}(x)\gamma_{5}\\ & + & 2\gamma_{5}S_{b}^{cb^{\prime }}(x)S_{d}^{\prime \;ba^{\prime }}(x)S_{u}^{ac^{\prime }}(x)\gamma_{5}+\gamma_{5}S_{d}^{ca^{\prime }}(x)S_{b}^{\prime \;bb^{\prime }}(x)S_{u}^{ac^{\prime }}(x)\gamma_{5}\\ & + & \gamma_{5}S_{d}^{ca^{\prime }}(x)S_{u}^{\prime \;ab^{\prime }}(x)S_{b}^{bc^{\prime }}(x)\gamma_{5}+\gamma_{5}S_{u}^{cb^{\prime }}(x)S_{b}^{\prime \;aa^{\prime }}(x)S_{d}^{bc^{\prime }}(x)\gamma_{5}\\ & + & 2\gamma_{5}S_{u}^{cb^{\prime }}(x)S_{d}^{\prime \;ba^{\prime }}(x)S_{b}^{ac^{\prime }}(x)\gamma_{5}-\gamma_{5}S_{u}^{cc^{\prime }}(x)\gamma_{5}Tr[S_{b}^{ab^{\prime }}(x)S_{d}^{\prime \;ba^{\prime }}(x)]\\ & - & \gamma_{5}S_{d}^{cc^{\prime }}(x)\gamma_{5}Tr[S_{u}^{ab^{\prime }}(x)S_{b}^{\prime \;ba^{\prime }}(x)]-4\gamma_{5}S_{b}^{cc^{\prime }}(x)\gamma_{5}Tr[S_{u}^{ab^{\prime }}(x)S_{d}^{\prime \;ba^{\prime }}(x)])\\ & + & \beta (2\gamma_{5}S_{b}^{ca^{\prime }}(x)\gamma_{5}S_{u}^{\prime \;ab^{\prime }}(x)S_{d}^{bc^{\prime }}(x)+2\gamma_{5}S_{b}^{cb^{\prime }}(x)\gamma_{5}S_{d}^{\prime \;ba^{\prime }}(x)S_{u}^{ac^{\prime }}(x)\\ & + & \gamma_{5}S_{d}^{ca^{\prime }}(x)\gamma_{5}S_{b}^{\prime \;bb^{\prime }}(x)S_{u}^{ac^{\prime }}(x)+2\gamma_{5}S_{d}^{ca^{\prime }}(x)\gamma_{5}S_{u}^{\prime \;ab^{\prime }}(x)S_{b}^{bc^{\prime }}(x)\\ & + & \gamma_{5}S_{u}^{cb^{\prime }}(x)\gamma_{5}S_{b}^{\prime \;aa^{\prime }}(x)S_{d}^{bc^{\prime }}(x)+2\gamma_{5}S_{u}^{cb^{\prime }}(x)\gamma_{5}S_{d}^{\prime \;ba^{\prime }}(x)S_{b}^{ac^{\prime }}(x)\\ & + & 2S_{b}^{ca^{\prime }}(x)S_{u}^{\prime \;ab^{\prime }}(x)\gamma_{5}S_{d}^{bc^{\prime }}(x)\gamma_{5}+2S_{b}^{cb^{\prime }}(x)S_{d}^{\prime \;ba^{\prime }}(x)\gamma_{5}S_{u}^{ac^{\prime }}(x)\gamma_{5}\\ & + & S_{d}^{ca^{\prime }}(x)S_{b}^{\prime \;bb^{\prime }}(x)\gamma_{5}S_{u}^{ac^{\prime }}(x)\gamma_{5}+2S_{d}^{ca^{\prime }}(x)S_{u}^{\prime \;ab^{\prime }}(x)\gamma_{5}S_{b}^{bc^{\prime }}(x)\gamma_{5}\\ & + & S_{u}^{cb^{\prime }}(x)S_{b}^{\prime \;aa^{\prime }}(x)\gamma_{5}S_{d}^{bc^{\prime }}(x)\gamma_{5}+2S_{u}^{cb^{\prime }}(x)S_{d}^{\prime \;ba^{\prime }}(x)\gamma_{5}S_{b}^{ac^{\prime }}(x)\gamma_{5}\\ & - & \gamma_{5}S_{u}^{cc^{\prime }}(x)Tr[S_{b}^{ab^{\prime }}(x)\gamma_{5}S_{d}^{\prime \;ba^{\prime }}(x)]-S_{u}^{cc^{\prime }}(x)\gamma_{5}Tr[S_{b}^{ab^{\prime }}(x)S_{d}^{\prime \;ba^{\prime }}(x)\gamma_{5}]\\ & - & \gamma_{5}S_{d}^{cc^{\prime }}(x)Tr[S_{u}^{ab^{\prime }}(x)\gamma_{5}S_{b}^{\prime \;ba^{\prime }}(x)]-4\gamma_{5}S_{b}^{cc^{\prime }}(x)Tr[S_{u}^{ab^{\prime }}(x)\gamma_{5}S_{d}^{\prime \;ba^{\prime }}(x)]\\ & - & S_{d}^{cc^{\prime }}(x)\gamma_{5}Tr[S_{u}^{ab^{\prime }}(x)S_{b}^{\prime \;ba^{\prime }}(x)\gamma_{5}]-4S_{b}^{cc^{\prime }}(x)\gamma_{5}Tr[S_{u}^{ab^{\prime }}(x)S_{d}^{\prime \;ba^{\prime }}(x)\gamma_{5}])\\ & - & \beta^{2}(2S_{b}^{ca^{\prime }}(x)\gamma_{5}S_{u}^{\prime \;ab^{\prime }}(x)\gamma_{5}S_{d}^{bc^{\prime }}(x)+2S_{b}^{cb^{\prime }}(x)\gamma_{5}S_{d}^{\prime \;ba^{\prime }}(x)\gamma_{5}S_{u}^{ac^{\prime }}(x)\\ & + & S_{d}^{ca^{\prime }}(x)\gamma_{5}S_{b}^{\prime \;bb^{\prime }}(x)\gamma_{5}S_{u}^{ac^{\prime }}(x)+2S_{d}^{ca^{\prime }}(x)\gamma_{5}S_{u}^{\prime \;ab^{\prime }}(x)\gamma_{5}S_{b}^{bc^{\prime }}(x)\\ & + & S_{u}^{cb^{\prime }}(x)\gamma_{5}S_{b}^{\prime \;aa^{\prime }}(x)\gamma_{5}S_{d}^{bc^{\prime }}(x)+2S_{u}^{cb^{\prime }}(x)\gamma_{5}S_{d}^{\prime \;ba^{\prime }}(x)\gamma_{5}S_{b}^{ac^{\prime }}(x)\\ & - & S_{d}^{cc^{\prime }}(x)Tr[S_{b}^{ba^{\prime }}(x)\gamma_{5}S_{u}^{\prime \;ab^{\prime }}(x)\gamma_{5}]-S_{u}^{cc^{\prime }}(x)Tr[S_{d}^{ba^{\prime }}(x)\gamma_{5}S_{b}^{\prime \;ab^{\prime }}(x)\gamma_{5}]\\ & - & 4S_{b}^{cc^{\prime }}(x)Tr[S_{d}^{ba^{\prime }}(x)\gamma_{5}S_{u}^{\prime \;ab^{\prime }}(x)\gamma_{5}])\}|\psi_{0}\rangle . \end{array}$$

Just by replacing the *d* quark with the *s* quark in the above equation, we get the correlation function for the $\Xi_{b}$ baryon. For $\Pi_{\Sigma_{b}}^{OPE}$ we get

(A.2) $$\begin{array}{ccc} \Pi_{\Sigma_{b}}^{OPE} & = & \frac{i}{2}\epsilon_{abc}\epsilon_{a^{\prime }b^{\prime }c^{\prime }}\int d^{4}xe^{ipx}\langle \psi_{0}|\{(\gamma_{5}S_{d}^{ca^{\prime }}(x)S_{b}^{\prime \;bb^{\prime }}(x)S_{u}^{ac^{\prime }}(x)\gamma_{5}\\ & + & \gamma_{5}S_{u}^{cb^{\prime }}(x)S_{b}^{\prime \;aa^{\prime }}(x)S_{d}^{bc^{\prime }}(x)\gamma_{5}+\gamma_{5}S_{u}^{cc^{\prime }}(x)\gamma_{5}Tr[S_{b}^{ab^{\prime }}(x)S_{d}^{\prime \;ba^{\prime }}(x)]\\ & + & \gamma_{5}S_{d}^{cc^{\prime }}(x)\gamma_{5}Tr[S_{u}^{ab^{\prime }}(x)S_{b}^{\prime \;ba^{\prime }}(x)])+\beta (\gamma_{5}S_{d}^{ca^{\prime }}(x)\gamma_{5}S_{b}^{\prime \;bb^{\prime }}(x)S_{u}^{ac^{\prime }}(x)\\ & + & \gamma_{5}S_{u}^{cb^{\prime }}(x)\gamma_{5}S_{b}^{\prime \;aa^{\prime }}(x)S_{d}^{bc^{\prime }}(x)+S_{d}^{ca^{\prime }}(x)S_{b}^{\prime \;bb^{\prime }}(x)\gamma_{5}S_{u}^{ac^{\prime }}(x)\gamma_{5}\\ & + & S_{u}^{cb^{\prime }}(x)S_{b}^{\prime \;aa^{\prime }}(x)\gamma_{5}S_{d}^{bc^{\prime }}(x)\gamma_{5}+\gamma_{5}S_{u}^{cc^{\prime }}(x)Tr[S_{b}^{ab^{\prime }}(x)\gamma_{5}S_{d}^{\prime \;ba^{\prime }}(x)]\\ & + & S_{u}^{cc^{\prime }}(x)\gamma_{5}Tr[S_{b}^{ab^{\prime }}(x)S_{d}^{\prime \;ba^{\prime }}(x)\gamma_{5}]+\gamma_{5}S_{d}^{cc^{\prime }}(x)Tr[S_{u}^{ab^{\prime }}(x)\gamma_{5}S_{b}^{\prime \;ba^{\prime }}(x)]\\ & + & S_{d}^{cc^{\prime }}(x)\gamma_{5}Tr[S_{u}^{ab^{\prime }}(x)S_{b}^{\prime \;ba^{\prime }}(x)\gamma_{5}])+\beta^{2}(S_{d}^{ca^{\prime }}(x)\gamma_{5}S_{b}^{\prime \;bb^{\prime }}(x)\gamma_{5}S_{u}^{ac^{\prime }}(x)\\ & + & S_{u}^{cb^{\prime }}(x)\gamma_{5}S_{b}^{\prime \;aa^{\prime }}(x)\gamma_{5}S_{d}^{bc^{\prime }}(x)+S_{d}^{cc^{\prime }}(x)Tr[S_{b}^{ba^{\prime }}(x)\gamma_{5}S_{u}^{\prime \;ab^{\prime }}(x)\gamma_{5}]\\ & + & S_{u}^{cc^{\prime }}(x)Tr[S_{d}^{ba^{\prime }}(x)\gamma_{5}S_{b}^{\prime \;ab^{\prime }}(x)\gamma_{5}])\}|\psi_{0}\rangle , \end{array}$$

where $S_{i}^{\prime }=CS_{i}^{T}C$ with i=u,d,s or *b* quark and the notation Tr[...] is used for the trace of gamma matrices. The correlation functions for *c*-baryons are obtained by the naive replacement b→c.

## References

[1] Prencipe E., Lange J.S., Blinov A. New spectroscopy with PANDA at FAIR: X, Y, Z and the F-wave charmonium states. arxiv:1512.05496.

[2] Biswas S. (2015). Measurement of the spark probability of a GEM detector for the CBM muon chamber (MuCh). Nucl. Instrum. Methods A.

[3] http://www.gsi.de/fair/experiments/CBM/index.e.html.

[4] http://www-panda.gsi.de/auto/phy/home.htm.

[5] Friman B. (2011). The CBM Physics Book: Compressed Baryonic Matter in Laboratory Experiments.

[6] Lutz M.F.M., PANDA Collaboration Physics performance report for PANDA: strong interaction studies with antiprotons. arxiv:0903.3905.

[7] Giacosa F. (2015). Non-conventional mesons at PANDA. J. Phys. Conf. Ser..

[8] Drukarev E.G., Levin E.M. (1990). The QCD sum rules and nuclear matter. 2. Nucl. Phys. A.

[9] Hatsuda T., Hogaasen H., Prakash M. (1991). QCD sum rules and the Okamoto–Nolen–Schiffer anomaly. Phys. Rev. Lett..

[10] Adami C., Brown G.E. (1991). Isospin breaking in nuclear physics: the Nolen–Schiffer effect. Z. Phys. A.

[11] Jin X.m., Furnstahl R.J. (1994). QCD sum rules for Lambda hyperons in nuclear matter. Phys. Rev. C.

[12] Jin X.M., Nielsen M. (1995). QCD sum rules for Sigma hyperons in nuclear matter. Phys. Rev. C.

[13] Suzuki K., Gubler P., Oka M. (2016). D meson mass increase by restoration of chiral symmetry in nuclear matter. Phys. Rev. C.

[14] Buchheim T., Hilger T., Kämpfer B. (2014). Heavy-quark expansion for *D* and *B* mesons in nuclear matter. EPJ Web Conf..

[15] Buchheim T., Kämpfer B., Hilger T. (2016). Algebraic vacuum limits of QCD condensates from in-medium projections of Lorentz tensors. J. Phys. G.

[16] Azizi K., Er N. (2014). Properties of nucleon in nuclear matter: once more. Eur. Phys. J. C.

[17] Azizi K., Er N., Sundu H. (2015). Positive and negative parity hyperons in nuclear medium. Phys. Rev. D.

[18] Hayashigaki A. (2000). Mass modification of D meson at finite density in QCD sum rule. Phys. Lett. B.

[19] Hilger T., Kampfer B. (2010). In-medium modifications of scalar charm mesons in nuclear matter. Nucl. Phys. B, Proc. Suppl..

[20] Wang Z.G., Huang T. (2011). In-medium mass modifications of the $D_{0}$ and $B_{0}$ mesons with the QCD sum rules. Phys. Rev. C.

[21] Azizi K., Er N., Sundu H. (2014). More about the *B* and *D* mesons in nuclear matter. Eur. Phys. J. C.

[22] Wang Z.G. (2011). Analysis of the $\Lambda_{Q}$ baryons in the nuclear matter with the QCD sum rules. Eur. Phys. J. C.

[23] Wang Z.G. (2012). Analysis of the $\Sigma_{Q}$ baryons in the nuclear matter with the QCD sum rules. Phys. Rev. C.

[24] Wang Z.G. (2012). Analysis of the doubly heavy baryons in the nuclear matter with the QCD sum rules. Eur. Phys. J. C.

[25] Thomas R., Hilger T., Kampfer B. (2007). Four-quark condensates in nucleon QCD sum rules. Nucl. Phys. A.

[26] Drukarev E.G., Ryskin M.G., Sadovnikov V.A. (2015). Nucleon QCD sum rules in instanton medium. J. Exp. Theor. Phys..

[27] Leinweber D.B. (1995). Nucleon properties from unconventional interpolating fields. Phys. Rev. D.

[28] Stein E., Gornicki P., Mankiewicz L., Schafer A., Greiner W. (1995). QCD sum rule calculation of twist-three contributions to polarized nucleon structure functions. Phys. Lett. B.

[29] Cohen T.D., Furnstahl R.J., Griegel D.K. (1991). From QCD sum rules to relativistic nuclear physics. Phys. Rev. Lett..

[30] Cohen T.D., Furnstahl R.J., Griegel D.K., Jin X.m. (1995). QCD sum rules and applications to nuclear physics. Prog. Part. Nucl. Phys..

[31] Furnstahl R.J., Jin X.m., Leinweber D.B. (1996). New QCD sum rules for nucleons in nuclear matter. Phys. Lett. B.

[32] Olive K.A., Particle Data Group (2014). Chin. Phys. C.

[33] Cohen T.D., Furnstahl R.J., Griegel D.K. (1992). Quark and gluon condensates in nuclear matter. Phys. Rev. C.

[34] Belyaev V.M., Ioffe B.L. (1983). Determination of the baryon mass and baryon resonances from the quantum-chromodynamics sum rule. Strange baryons. Sov. Phys. JETP.

[35] Ioffe B.L. (2006). QCD at low energies. Prog. Part. Nucl. Phys..

[36] Thomas A.W., Shanahan P.E., Young R.D. (2012). Nuovo Cimento C.

[37] Dinter S., Drach V., Jansen K. (2011). Dark matter search and the scalar quark contents of the nucleon. Int. J. Mod. Phys. E.

[38] Aliev T.M., Azizi K., Ozpineci A. (2009). Radiative decays of the heavy flavored baryons in light cone QCD sum rules. Phys. Rev. D.

